# Fulminant Pneumonia Due to Reactivation of Latent Toxoplasmosis in a Cat—A Case Report

**DOI:** 10.3390/pathogens13010007

**Published:** 2023-12-20

**Authors:** Simone A. Fietz, Thomas Grochow, Gereon Schares, Tanja Töpfer, Romy M. Heilmann

**Affiliations:** 1 Institute of Veterinary Anatomy, Histology and Embryology, College of Veterinary Medicine, Leipzig University, DE-04103 Leipzig, Germany; simone.fietz@vetmed.uni-leipzig.de (S.A.F.); th.grochow@web.de (T.G.); 2 National Reference Laboratory for Toxoplasmosis, Institute of Epidemiology, Friedrich-Loeffler-Institute, Federal Research Institute for Animal Health, DE-17493 Greifswald-Insel Riems, Germany; gereon.schares@fli.de; 3 Department for Small Animals, College of Veterinary Medicine, University of Leipzig, DE-04103 Leipzig, Germany; tanja.toepfer@kleintierklinik.uni-leipzig.de

**Keywords:** *Toxoplasma gondii*, infective pneumonia, immunosuppression, zoonosis, cat, latent toxoplasmosis

## Abstract

*Toxoplasma (T.) gondii* is an obligate intracellular parasite with felids, including domestic cats, as definitive hosts. In immunocompetent individuals, *T. gondii* infection is usually asymptomatic. However, under immunosuppression, it may have severe pathological impacts, which often result from the reactivation of a chronic infection. In this case study, a 21-month-old female domestic shorthair cat—diagnosed with primary immune-mediated hemolytic anemia three months prior and treated with cyclosporine and prednisolone—presented with acute tachypnea, dyspnea, diarrhea, and anorexia. Thoracic radiography suggested severe pneumonia. Testing for *Mycoplasma* spp., *Anaplasma* spp., *Ehrlichia* spp., and lungworm infection was negative. Serology for *T. gondii* revealed seroconversion of IgG, but not of IgM, indicating previous exposure to *T. gondii*. The cat remained stable but tachypneic for three days, followed by an acute onset of dyspnea and clinical deterioration, after which euthanasia was elected. Numerous protozoa were present in a postmortem transtracheal bronchoalveolar lavage and fine-needle aspiration of the lung. Microsatellite typing classified the extracted DNA as *T. gondii* type II variant TgM-A. This case demonstrates that *T. gondii* reactivation, leading to fulminant pneumonia, can be a sequela of immunosuppressive treatment in cats and should, therefore, be considered as a differential diagnosis in immunosuppressed cats with acute-onset respiratory signs. Rapid diagnosis may prevent fatal consequences.

## 1. Introduction

Autoimmune conditions are common in companion animals, including cats, and can either involve several different organ systems or present a multicentric condition such as systemic lupus erythematosus [1]. Differences in the frequency of occurrence, underlying etiologies, and pathomechanisms vary between dogs and cats, and species-specific differences in the immune response—particularly the T-helper-cell balance—might contribute to some differences between both species [2]. Immune-mediated hemolytic anemia (IMHA) is also common in cats [3], requiring the exclusion of non-immune-mediated causes [4,5,6,7,8,9] as well as infectious (e.g., hemotrophic *Mycoplasma* species) and neoplastic etiologies (e.g., lymphoma) for a diagnosis of primary autoimmune hemolytic anemia (AIHA) [10]. The mainstay of AIHA treatment is immunosuppression and the prevention of complications such as thrombosis and thromboembolism [3,11,12]. Secondary infections are also a concern in any natural or iatrogenic immunosuppressed patient [13,14], and this can include infectious bacterial or protozoal pneumonia and the development of septicemia with potentially fatal outcomes [15].

*Toxoplasma (T.) gondii* is an obligate intracellular *protozoan* parasite with the cat as its definitive host and all warm-blooded animals as intermediate hosts [16]. It is globally distributed and causes the zoonosis toxoplasmosis. Two recent systematic analyses found that its global seroprevalence is estimated at 26% in humans [17] and 35% in domestic cats [18]. *T. gondii* is the only species in the genus *Toxoplasma*. In Europe, most genotypes observed belong to three clonal lineages (types I, II, and III), with type II representing the genotype of the vast majority of isolates. *T. gondii* has several developmental stages during its life cycle [19]. Sexual reproduction of the parasite occurs in the intestine of the definitive host, with the resulting oocysts being exclusively excreted in the feces. Oral infection results in rapid asexual multiplication of the acute (tachyzoite) stage, followed by the infection of all tissues via the blood, lymph and peritoneal fluid [20]. In chronic stages of the disease, intracellular tissue cysts with bradyzoites are formed, which have a reduced metabolism and may persist *throughout the host’s lifetime* [21]. In adult healthy and immunocompetent individuals, *T. gondii* infection is usually asymptomatic. However, in people who are immunosuppressed due to autoimmune disease treatment or underlying disease (e.g., with human immunodeficiency virus), *T. gondii* infection may have severe pathological impacts, which most often result from the reactivation of a chronic *T. gondii* infection [22,23,24,25]. Similarly, localized or disseminated toxoplasmosis in cats has been associated with immunodeficient states following treatment with immunosuppressant medication [26,27,28,29] or retrovirus infection [30,31]. Corresponding clinical signs include fever, loss of appetite, weight loss, lethargy, neurological signs (e.g., tremors or seizures), pneumonia, uveitis, retinitis, and hepatitis. Given the broad spectrum of clinical manifestations, diagnosis of feline toxoplasmosis is often challenging and contributes to fatal outcomes if cases remain undiagnosed.

This case report documents the reactivation of latent toxoplasmosis and its clinical manifestation in the respiratory system following immunosuppressive treatment in a cat.

## 2. Case Report

A 21-month-old female domestic shorthair cat (3.3 kg) presented with acute onset of tachypnea and dyspnea at home. The cat also had acute diarrhea and anorexia in the two days prior to presentation and was progressively lethargic. The pertinent patient history included a primary IMHA (AIHA) that was diagnosed three months earlier. To treat the AIHA, the cat received cyclosporin (Atopica^®^, Elanco, Bad Homburg, Germany; at 5 mg/kg PO q12h) monotherapy after combination therapy with prednisolone (started at 1.4 mg/kg PO q12h) was tapered (dose reduction by 25% every 2–3 weeks) and discontinued two weeks before presentation. The cat was strictly housed indoors, had no obvious concurrent conditions, and received no other medications or supplements. Vaccinations were not current, but the cat had been regularly dewormed. Retrovirus testing (feline leukemia virus and feline immunodeficiency virus) was negative at the time of IMHA diagnosis.

Upon physical examination, the cat was quiet, alert, and responsive but experienced distress with handling. The heart rate was 160/min (no pulse deficit), and the respiratory rate was 68/min (forceful breathing with abdominal push); the rectal body temperature could not be measured. Oral mucous membranes were pale, with a capillary refill time of <2 s. Sneezing, nasal discharge, or spontaneous coughing were not noted. Palpation of the abdomen and peripheral lymph nodes was unremarkable.

**Figure 1:**
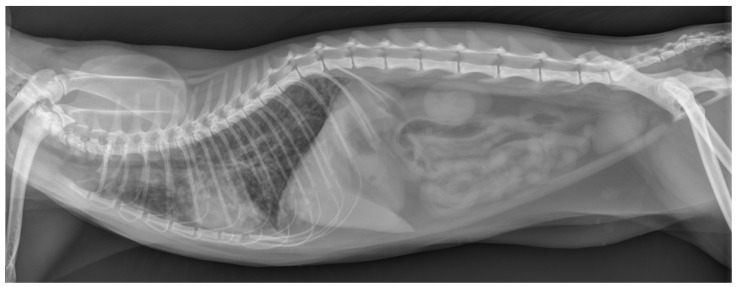
Thoracic radiograph at the time of presentation. This laterolateral view shows a generalized mixed bronchointerstitial to alveolar lung pattern, resulting in a decreased delineation of the cardiac silhouette.

Abdominal ultrasonography revealed no abnormalities of the liver, spleen, kidneys, adrenal glands, pancreas, liver, gall bladder, stomach, and intestines, but mild mesenteric lymphadenopathy likely reflected a reactive change. A laterolateral thoracic radiograph was performed and showed a generally reduced opacity of the lung fields with a mixed bronchointerstitial to alveolar lung pattern, a poorly delineated cardiac silhouette and a mildly increased vertebral heart size (VHS) of 9.4 (normal right lateral VHS: <8.1) (Figure 1).

Hematology revealed normal erythrogram findings (hematocrit: 37.3%, reference interval (RI): 30.3–52.3%; mean corpuscular volume: 38.0 fL, RI: 35.9–53.1 fL; mean corpuscular hemoglobin: 13.4 pg, RI: 11.8–17.3 pg; reticulocyte count: 13.7 × 10^9^/L, RI: <50 × 10^9^/L). In addition, there was a marked leukopenia (0.81 × 10^9^/L, RI: 2.87–17.02 × 10^9^/L) characterized by mature neutropenia (0.13 × 10^9^/L, RI: 1.48–10.29 × 10^9^/L) and mild thrombocytopenia (automated count: 137 × 10^9^/L, manual count: 118 × 10^9^/L; RI: 151–600 × 10^9^/L). The serum biochemistry profile showed a high-normal blood glucose concentration (8.2 mmol/L, RI: 3.9–8.2 mmol/L), low-normal potassium concentration (3.5 mmol/L, RI: 3.4–4.6 mmol/L), and minimal hyperchloremia (sodium-corrected chloride: 120.2 mmol/L, RI: 107–120 mmol/L), while all other parameters were within the corresponding normal reference intervals. The patient-side NT-proBNP (N-terminal pro-brain natriuretic peptide) test (feline proBNP SNAP^®^, Idexx Laboratories, Kornwestheim, Germany) was normal. Fecal flotation/sedimentation and the Baerman technique for lungworm detection were negative. Moreover, polymerase chain reaction (PCR)-based testing for feline hemotrophic mycoplasmosis (*M. hemofelis*, *Cand. M. hemominutum*, and *Cand. M. turicensis*) and serology for *Anaplasma* spp. and *Ehrlichia canis* were all negative. Serological testing for *T. gondii* using an indirect immunofluorescence assay (IDEXX Laboratories, Kornwestheim, Germany) was negative for immunoglobulin M (IgM) but positive for IgG at dilutions >1:1024.

More invasive diagnostics comprising a tracheobronchoscopy with bronchoalveolar lavage (for cytology and clinical microbiology) under general anesthesia were considered to evaluate important differential diagnoses further. However, these could not be performed given the overall clinical condition of the cat.

**Figure 2:**
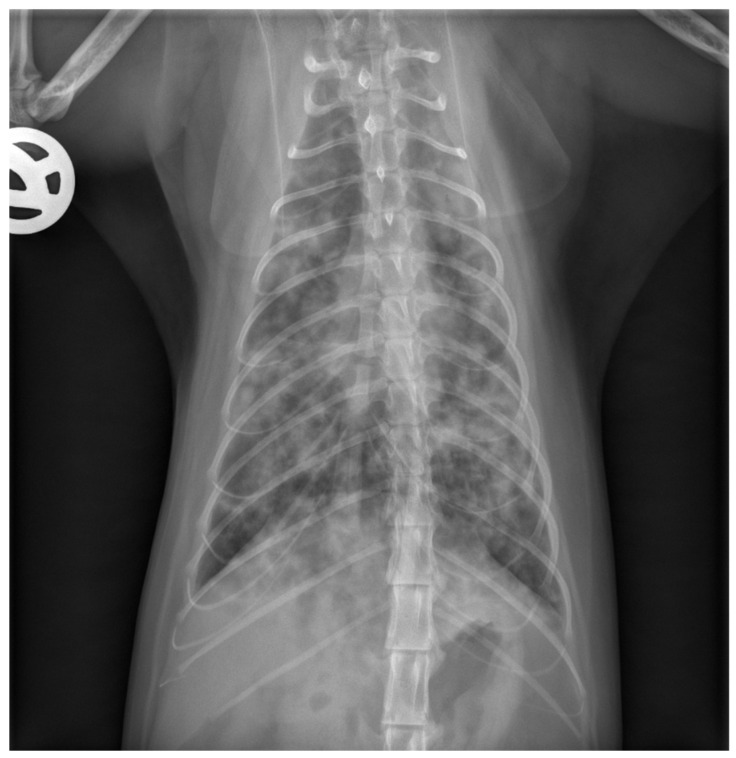
Repeat thoracic radiograph after 3 days of hospitalization. This ventrodorsal view shows a progressive bronchointerstitial to alveolar lung pattern compared to the initial radiographs obtained at the time of presentation.

The cat was hospitalized in the intensive care unit and received supplemental oxygen (oxygen cage, 40% O_2_), intravenous crystalloid fluid support (Ringer’s Acetate solution), bronchodilation with theophylline (Euphylong^®^ Injectable solution, Altana Pharma, Konstanz, Germany), amoxicillin/clavulanate (AmoxiClav^®^, Hikma, Munich, Germany) antimicrobial treatment, and empirical deworming with fenbendazole (Panacur^®^, MSD-Intervet, Unterschleißheim, Germany), and was closely monitored for any changes in its respiratory rate and effort or other complications. Carefully weighing the benefits of the current treatment strategy against the risks of AIHA relapse and the working diagnosis of secondary infectious pneumonia, cyclosporine treatment was decided to be continued. Given the severe neutropenia and lack of significant clinical improvement within 24 h, clindamycin (Cleorobe^®^, Zoetis, Berlin, Germany) was added to the treatment plan. The cat remained stable but tachypneic for three days, followed by an acute onset of dyspnea on day four of hospitalization. Repeat thoracic radiographs (only one image plane due to the clinical instability of the cat) revealed mildly progressive changes (Figure 2). The cat acutely decompensated, and humane euthanasia was elected by the owner.

**Figure 3:**
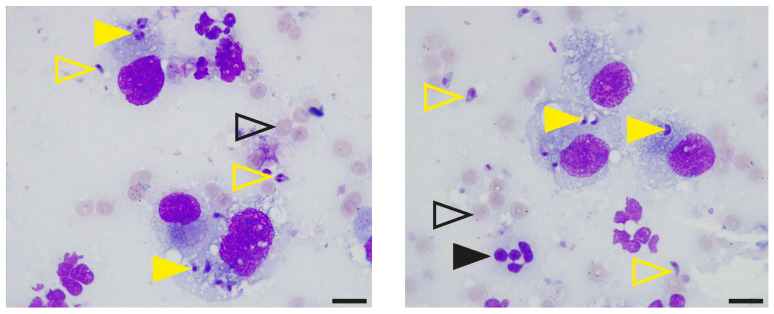
Identification of protozoal structures in the feline bronchoalveolar lavage fluid. Samples of bronchoalveolar lavage fluid and fine-needle aspiration of the lung were centrifuged onto glass slides, stained with Diff-Quik and examined using a Leica DM 750 light microscope equipped with a 100× oil objective (NA 1.25) and a Leica ICC50W camera (Leica, Wetzlar, Germany). Open yellow arrowheads indicate extracellular protozoa, and closed arrowheads in yellow point to protozoa within alveolar macrophages. Open black arrowheads show erythrocytes, and closed arrowheads in black indicate neutrophils. Scale bars, 10 µm.

A postmortem transtracheal bronchoalveolar lavage (BAL) and fine-needle aspiration (FNA) of the lung were performed. Cytology of the BAL fluid and FNA samples, stained with Diff-Quik, revealed numerous protozoal structures that were distributed extracellularly as well as within alveolar macrophages (Figure 3). Microsatellite typing of the protozoal DNA extracted from BAL fluid and lung FNA samples classified the isolate as *T. gondii* type II variant TgM-A (Table 1).

## 3. Discussion

We report a feline case of fulminant pneumonia, presumably due to the reactivation of latent *T. gondii* infection.

Based on a complete clinical evaluation and diagnostic work-up three months prior to presentation at the clinic with acute-onset respiratory signs, the cat was diagnosed with primary IMHA (AIHA). Treatment for primary IMHA consisted of immunosuppressant medications, using a combination of cyclosporine and prednisolone in a top-down treatment approach. Hematology revealed normal erythrogram findings (i.e., hematocrit, mean corpuscular volume, mean corpuscular hemoglobin, reticulocyte count), indicating that the primary IMHA was well controlled. However, the cat was in an immunosuppressed state given the treatment of the underlying disease and the development of marked hematologic abnormalities (marked leukopenia, mature neutropenia, and mild to moderate thrombocytopenia).

Diagnostic imaging revealed a normal appearance of abdominal organs (i.e., liver, spleen, kidneys, adrenal glands, pancreas, liver, gall bladder, stomach, and intestines) but marked alterations of the intrathoracic respiratory system (i.e., all lung fields). Specifically, thoracic radiographs at the time of presentation showed a mild generalized alveolar lung pattern with air-bronchograms superimposed on the cardiac silhouette and a mildly increased vertebral heart size, suggesting either pulmonary edema (cardiogenic vs. non-cardiogenic) or pneumonia (bacterial, parasitic, or other etiologies), but less likely diffuse infiltrative neoplasia (e.g., lymphoma, mast cell tumor). The NT-proBNP test was normal, supporting the suggestion that a non-cardiogenic etiology was more likely.

Vector-borne pathogens *(M. hemofelis*, *Cand. M. hemominutum*, and *Cand. M. turicensis*; *Anaplasma* spp. and *Ehrlichia canis)* and lungworm infection were ruled out as possible causes based on negative testing. However, serology revealed a high *T. gondii*-specific IgG titer (>1:1024) but no IgM titer, indicating a previous exposure to *T. gondii* that resulted in latent toxoplasmosis. Two similar cases with predominantly or exclusively IgG positivity were described in Australia [34], whereas a newly acquired infection was presumed in a cyclosporine-treated cat with fatal toxoplasmosis reported in South Africa [35]. The cat reported here was not given a raw meat-based diet (RMBD) for months prior to presentation, which could have been the source of an acute *T. gondii* infection. However, as it had been reported to have dug in the soil and licked stones on the owner’s patio when first presented for IMHA (presumably as a physiological compensatory response to the marked anemia), a recent infection via *T. gondii*-contaminated soil cannot be entirely excluded. While this would be expected to concur with a positive IgM titer, the antibody response may have been delayed in the face of iatrogenic immunosuppression. At the time of primary IMHA diagnosis, the cat had received blood products as an emergency stabilization measure. However, blood transfusion (A-type donor used based on blood typing) is an unlikely cause of *T. gondii* infection.

Direct tests (i.e., cytology and microsatellite genotyping) were utilized to confirm the suspicion of toxoplasmosis, specifically its manifestation in the respiratory system. Microscopic examination of Diff-Quik-stained BAL fluid and FNA cytology sections revealed the presence of numerous protozoal structures, substantiating the suspicion of respiratory toxoplasmosis. Microsatellite analysis confirmed the presence of *T. gondii* in BAL fluid material and FNA cytology sections and further classified the isolate as *T. gondii* type II strain, which is the *predominating strain* in humans and domestic animals in Europe and North America [36]. Altogether, these results confirmed the diagnosis of infectious pneumonia in this cat and identified the protozoal organism *T. gondii* as the primary pathogen. In immunocompromised people, reactivation of *T. gondii* most often manifests as encephalitis and, less frequently, as pneumonia, retinochoroiditis, or disseminated systemic disease [15,25,37]. Given the isolation of a common *T. gondii* strain in the geographic location of the reported feline case, an impaired clearance of this pathogen by the infected yet immunocompromised host more likely explains the fulminant disease course and fatal outcome than an increased pathogenicity of the organism. Our findings confirm previous reports in cats, in which reactive or acute toxoplasmosis was diagnosed following treatment with immunosuppressant medication, e.g., prednisolone and cyclosporine therapy [26,27,28,29,34,35].

## 4. Conclusions

*T. gondii* infection has a broad spectrum of clinical manifestations. This case report shows that *T. gondii* reactivation, leading to fulminant pneumonia, can be a sequela of immunosuppressive treatment in cats. Hence, *T. gondii* infection and its manifestation in the respiratory system should be considered in the differential diagnosis list for cats showing acute-onset respiratory signs following treatment with immunosuppressants or retrovirus infection. A rapid diagnosis of this complication can potentially decrease the risk of fatal outcomes.

## Figures and Tables

**Table 1:** Microsatellite (MS) analysis of bronchoalveolar lavage fluid samples and fine-needle aspirates of the lung. DNA was extracted from cells and tissue using a NucleoSpin Tissue Mini kit for DNA (Machery-Nagel, Germany) and was analyzed via microsatellite typing. DNA was amplified using a multiplex PCR [25] including 15 unlinked MS markers, eight typing markers (TUB2, W35, TgM-A, B18, B17, M33, IV.1, XI.1) and seven fingerprinting markers (M48, M102, N83, N82, AA, N61, N60). Fingerprinting markers display a high level of polymorphism within the clonal lineages type I, type II, and type III [32]. Primers were used at a concentration of 0.2 pmol/µL. The only divergence from the original method was that in the case of M102, AA, and N60, and the fluorophore Atto 550 was used instead of NED to label amplicons during multiplex PCR. Samples were applied as duplicates. Water was used as a negative control, and *T. gondii* RH, Me49, and NED—corresponding to type I, II, and III strains, respectively—served as positive controls. Typing followed recently published guidelines, and the results were numerically corrected as recommended [33].

Sample	*T. gondii* Variant														
	6-FAM N61	6-FAM B18	6-FAM M33	6-FAM M48	6-FAM TUB2	6-FAM N83	6-FAM XI.1	HEX N82	HEX TgM-A	HEX W35	HEX IV.1	HEX B17	NED N60	NED M102	NED AA
SAMPLE	103	158	169	209	289	312	356	121	209	242	274	336	140	174	277
SAMPLE	103	158	169	209	289	312	356	121	209	242	274	336	140	174	277
Negative control	NA	NA	NA	NA	NA	NA	NA	NA	NA	NA	NA	NA	NA	NA	NA
RH	87	160	169	209	291	306	358	119	209	248	274	342	145	166	265
Me49	91	158	169	215	289	310	356	111	207	242	274	336	142	174	265
NED	91	160	165	209	289	312	356	111	205	242	278	336	147	190	269

NA: not applicable.

## Data Availability

Data and information about this case are available from the corresponding author upon reasonable request.
